# Supplementary material for: Comparison of medical students’ perceptions of patient safety: Focusing on simulation training using a high-fidelity simulator
Source: PLoS One. 2024 Jul 18;19(7):e0304883. doi: 10.1371/journal.pone.0304883 (PMC11257313; doi:10.1371/journal.pone.0304883)
Supplement: Attachment — Submitted filename: Response to Reviewers.docx [file pone.0304883.s001.docx]

Dear Editor:

We would like to re-submit the manuscript titled, “**Comparison of medical students’ perceptions of patient safety: Focusing on simulation training using a high-fidelity simulator**”. The manuscript number is **PONE-D-22-35534**. We thank you and the reviewers for your thoughtful suggestions and insights. The manuscript has benefited from these insightful suggestions. We look forward to working with you and the reviewers to move this manuscript closer to publication in PLOS ONE. The manuscript has been rechecked, and the necessary changes have been made in accordance with the reviewer’s suggestions. The responses to all comments have been prepared and are attached herewith.

Thank you for your consideration. We look forward to hearing from you.

Sincerely,

Hyun Joo Jung

**Reviewer 1**

1. First, I would like more background on the simulation used in safety courses. We know it enables practice in a safe environment, but I would like you to Focus on the immediate connection with your text.

**Response:** In Korea, more research is being conducted on patient safety education for nursing students. We have included some information on this and cited the relevant studies. In addition, some sentences have been modified, focusing on the natural connection of the text. (Revised manuscript page 3, line 10-15)

2. Not explicit, the introduction is too long when a whole manuscript is considered.

**Response:** Thank you for pointing this out. The background of this study was explained in detail so that even those who are not familiar with the subject can easily understand it. However, we agree that the introduction could be shortened, and we have revised it accordingly.

3. Additionally, there is no detailed description of the research group or the setting; the results are mixed with the methodology.

We need more details on the scenarios run in the sessions and how they were implemented- all eight students a once-at-one scenario. I would not describe debriefing as feedback; it has a broader function- please list which debriefing method was used and what were the main points of debriefing. We have no doubts that a qualified person runs it in medical school—more details on the process. To few details are given on the questionnaires used.

The results are very basic, it is a very simple pre-post comparison. We have no validation of the questionnaire. Please re-think the calculations and how they apply to the clinical actions. There may be more data to work on.

**Response:** We agree with your suggestions. Thus, we added more details on the simulation training program in the form of figures. We hope that these additions clarify how our training program was run.

In terms of more data, the only available data that we had were the pre-post survey data that investigated students’ perceptions. However, we agree that this can be taken further, and we are preparing a long-term observational study with more medical school students. We will design a study with your insightful suggestions in mind. We have added to list this as a limitation. (Revised manuscript page 3, line 10-15)

4. Conclusions are just repetitions of the results.

**Response:** We have revised or deleted the repetitive text (Revised manuscript page 7-9). In the conclusion section, we now discuss what we want to emphasize and what the future direction of education should be.

**Reviewer 2**

1. In general an English language editing is required to decrease the word count in certain parts'

**Response:** As per your suggestion, we have consulted Editage, an English language editing company, to correct all grammar- and language-related errors and reduce the word count.

2. Introduction: no need for too much details on benefit of simulation education

**Response:** We agree and have revised the introduction to briefly explain the benefits of simulation education. (Revised manuscript page 3, line 62-70)

3. Method: how you educate the students , elaborate more on the content of their training , you only mentioned the process

**Response:** We added more details of the simulation training program in the form of figures to provide more details of the training program. (Revised manuscript page 5, line 104-108)

4. Results: a. No 4 and 15 , 19 in table 1 the same data reading noticed in pre and post survey but the p value show highly significant????

b. the table headings is it P value or t value

c. Total in the table is not clear is it the sum , average??? you need to clarify more

d. No need to repeat the result of the table in the written text just concentrate on the main changes

**Response:** Thank you for pointing out these issues. Our point-by-point responses are as follows:

a & b: We made a mistake in the process of making the table, for which we apologize, and we have now revised it. The previous table did not contain p-values, which may have caused confusion. Therefore, we have now added them to the table. In the previous table, the * mark (*p*-value<0.05) for No. 4, 15, and 19 was an error. The significant questions were No. 3, 20, 24, 25, 26, and 30. We have revised the table to clarify our results.

c. In the previous table, “Total” referred to the average value of the sub-variable question items. We have revised the terminology used to make this clearer.

d. As per your suggestion, we have removed repetitive information from the text.

(Revised manuscript page 7-9, In Results)

5. In Discussion it is not clear weather your students have repeated training or it was single training opportunity (2nd paragraph)

**Response:** We conducted one single training session, and we have specified this in the discussion section. (Revised manuscript page 9, In Discussion, 2^nd^ paragraph)

**Editor**

1. Please ensure that your manuscript meets PLOS ONE's style requirements, including those for file naming. The PLOS ONE style templates can be found at

https://journals.plos.org/plosone/s/file?id=wjVg/PLOSOne_formatting_sample_main_body.pdf and

<https://journals.plos.org/plosone/s/file?id=ba62/PLOSOne_formatting_sample_title_authors_affiliations.pdf>

**Response:** We have re-checked the formatting requirements and amended the document to adhere to them, including the file name.

2. We note that you have indicated that data from this study are available upon request. PLOS only allows data to be available upon request if there are legal or ethical restrictions on sharing data publicly. For more information on unacceptable data access restrictions, please see http://journals.plos.org/plosone/s/data-availability#loc-unacceptable-data-access-restrictions.

In your revised cover letter, please address the following prompts:

a) If there are ethical or legal restrictions on sharing a de-identified data set, please explain them in detail (e.g., data contain potentially sensitive information, data are owned by a third-party organization, etc.) and who has imposed them (e.g., an ethics committee). Please also provide contact information for a data access committee, ethics committee, or other institutional body to which data requests may be sent.

b) If there are no restrictions, please upload the minimal anonymized data set necessary to replicate your study findings as either Supporting Information files or to a stable, public repository and provide us with the relevant URLs, DOIs, or accession numbers. For a list of acceptable repositories, please see <http://journals.plos.org/plosone/s/data-availability#loc-recommended-repositories>.

**Response:** Since we conducted a study on a small number of people, all survey data were written on paper. Therefore, we do not have URLs or DOIs that contain uploaded online data. The original data have been stored in the data storage room of the ACE center at Ajou University Medical School with the approval of Ajou University IRB **(Approved No. AJIRB-SBR-SUR-21-624)**. The storage room is always locked. One can request the data at any time through ‘data access committee’ (the following contact information). If PLOS ONE requests the original data, it can be scanned and sent to you. In addition, since the original data are in Korean, we will translate the questionnaire into English and attach it as a supplement.

**[Data Access Committee]**

Ji Eun Lee

- Tel. : 82-10-3929-7011
- E-mail : [jieun920733@gmail.com](mailto:jieun920733@gmail.com)
- Hyperlink : Instagram @nueiz.e

Ji Hye Yu

- Tel. : 82-31-219-4092
- E-mail : blue8106@aumc.ac.kr
- Hyperlink : no available hyperlink

Su Kyung Lee

- Tel. : 82-31-219-4463
- E-mail : esukyung71@gmail.com
- Hyperlink : no available hyperlink

Jang Hoon Lee

- Tel. : 82-31-219-5167
- E-mail : neopedlee@aumc.ac.kr
- Hyperlink : no available hyperlink

Hyun Joo Jung

- Tel. : 82-31-219-5160
- E-mail : [free1109@ajou.ac.kr](mailto:free1109@ajou.ac.kr)
- Hyperlink : no available hyperlink

**[Ethics Committee]**

Ajou University IRB

- Tel. : 82-31-219-5569
- E-mail : aumc_qa@aumc.ac.kr
- Hyperlink : https://eirb.ajoumc.or.kr/
