# Supplementary material for: Comparison of medical students’ perceptions of patient safety: Focusing on simulation training using a high-fidelity simulator
Source: PLoS One. 2024 Jul 18;19(7):e0304883. doi: 10.1371/journal.pone.0304883 (PMC11257313; doi:10.1371/journal.pone.0304883)
Supplement: Attachment — Submitted filename: Response_to_Reviewers.docx [file pone.0304883.s002.docx]

Dear Editor and Reviewers:

Once again, we thank you and the reviewers for your thoughtful suggestions and insights. Below, please find our point-by-point responses to the reviewers’ comments.

We look forward to any further input you may have for us.

Sincerely,

Hyun Joo Jung

**Reviewer 3**

1. They have addressed all the concerned issues, they have shortened the introduction, revised the statistical analysis, I think, it should be accepted.

**Response:** Thank you for your affirmative comment. We found the comments shared by the reviewers from the previous round of review to be valid and valuable, and revised our manuscript accordingly.

**Reviewer 4**

1. Having read the previous reviewer comments, I feel that the introduction remains too long and is not germane to the study itself. The introduction should focus on simulation safety education literature and how this study would contribute to the literature/answer an unanswered question. Since simulation education like this has been provided for many, as has patient safety education, there needs to be a clearer delineation of the need for this work. Page 4, lines 73-75 are repetitive of a point made in the preceding paragraph; one or the other can be eliminated. Apropos to better delineation of the need for the study, since this is a topic that has been explored before and has already been shown in Korea in nursing students, why should simulation education affect on medical students be different?

**Response:** Thank you for your insightful comment. In Korea, because the roles of doctors and nurses within hospitals are clearly different, it was believed that there should be differences in education depending on each job group. The duties of doctors and nurses are strictly divided, and doctors have the full and final responsibility for patient safety and management. Therefore, patient safety education for medical students and establishing awareness of patient safety through this education are very important in the medical student curriculum. In addition, since medical schools in Korea have not yet established a school-level curriculum, there is a need to try various methods and determine educational directions. In this regard, the manuscript was slightly modified [Introduction, paragraph 3]. Regarding lines 73–75 on page 4, the manuscript was revised because we agree with the opinion that there is some overlap with the previous paragraph.

2. Both Figures 1 and 2 do not appear in the copy of the manuscript that I am able to access. Their legends do, as does the entirety of Table 1, so I am unable to state specifically anything regarding their effectiveness/appropriateness. In the Design and Setting, very little demographic data is presented (28 males, 12 females) -- what else is known about the survey/study participants? Ages? Experiences (either work, research, or clinical) that may have affected their answers? More information is needed.

**Response:** Thank you for your helpful comment. Figure 1 illustrates the learning objectives according to the stages of the simulation program and Figure 2 summarizes the process of the simulation program. We believe that there was an error when resubmitting the manuscript; our apologies. We will upload it again.

The students who participated in this study are fifth-year students taking the same curriculum at the medical school. We can provide basic data such as the age of all students who participated in this study and whether they majored in other fields before entering medical school. However, in this study, the survey was conducted anonymously to obtain honest answers from students, so demographic data and the impact of survey answers could not be analyzed.

Specifically, the 40 students who participated in this study were all in their 20s, ranging in age from 22 to 29 years. Among them, one person had a degree in another major (i.e., computer engineering) before entering medical school. Additionally, these students had no other research experience in the field of basic medicine or sub-internship experience before participating in this study.

3. Under Simulation Program Setting (page 5, lines 113-4), the statement is made that debriefing was provided to provide feedback on the learners' knowledge, skills, and attitude. This is not enough information, and does not address adequat5ely previous reviewer concerns. was a specific tool utilized to ensure that all the objectives of the simulation were achieved? did the students switch roles (they were paired up, but it does not say if they had different roles and then did the scenario again after switching roles)?

**Response:** Thank you for your valuable comment. We conducted debriefing using the “PEARLS healthcare debriefing tool.” Therefore, we did not proceed with the simulation program by switching roles after the debriefing session. We simply surveyed the students about how they felt before and after the simulation training. However, we think that it is necessary to conduct a simulation again after the debriefing to determine how the students’ perceptions level has changed. Therefore, we hope that future research focusing on this topic can be conducted.

4. Of the 30 questions, only 6 had statistically signfiicant changes in responses, 3 in attitude and 3 in self-efficacy. Average score across 24 attitude items did not change. Average score across the 6 self-efficacy items did, which was the predominant driver of the overall average over 30 questions of a statistically significant average score. I find the limited number of items that showed change less convincing of the effectiveness of the education than the authors do, and feel that the conclusions are too broad compared to the limited effects demonstrated in the results section.

**Response:** Thank you for your insightful comment. We agree with some of your perspectives. Therefore, we have revised the manuscript by mentioning the aspects you have pointed out in the paragraph where we present our limitations [Discussion, paragraph 4]. The number of items showing significant changes is small, which may seem unconvincing. However, it can be seen that just one training session can sufficiently increase students’ self-efficacy. Attitudes, meanwhile, seem difficult to change with one-time training. Therefore, we believe that repetitive training is necessary, and as mentioned in the paper, we are preparing a study to examine the effects of long-term training.
