# Supplementary material for: Comparison of medical students’ perceptions of patient safety: Focusing on simulation training using a high-fidelity simulator
Source: PLoS One. 2024 Jul 18;19(7):e0304883. doi: 10.1371/journal.pone.0304883 (PMC11257313; doi:10.1371/journal.pone.0304883)
Supplement: Attachment — Submitted filename: Response_to_Reviewers.docx [file pone.0304883.s003.docx]

Dear Editor:

We would like to re-submit the manuscript titled, “**Comparison of medical students’ perceptions of patient safety: Focusing on simulation training using a high-fidelity simulator**”. The manuscript number is **PONE-D-22-35534-R2**. We thank you and the reviewers for your thoughtful suggestions and insights. The manuscript has benefited from these insightful suggestions. We look forward to working with you and the reviewers to move this manuscript closer to publication in *PLOS ONE*. The manuscript has been rechecked, and the necessary changes have been made in accordance with the reviewer’s suggestions. The responses to all comments have been prepared and are attached herewith.

Thank you for your consideration. We look forward to hearing from you.

Sincerely,

Hyun Joo Jung

**Reviewer 5**

1. The manuscript seems to have been revised a lot, according to previous reviewer comments. However, in my opinion, to reflect their comments, further revision would be necessary. First, the necessity of this study should be mentioned in terms of patient safety and simulating training together. In the introduction, the authors seem to be dealing with the necessity of this study in terms of patient safety and simulating training separately. This discrepancy weakens the necessity of this study.

**Response:** Thank you for your valuable comment. We reviewed the introduction again. In response to your comment, we have added brief information about the link between patient safety and simulation education. [Introduction, paragraph 4]

2. Second, Figure 2 does not reflect the process of the simulation program’. It looks like the process of this study. Under the title'simulation program setting’, the authors do not carefully use the terms'simulation program' and'study' differently, which confuses readers about the study between the traning programs. Fig. 2 should be included under the title “Design and Setting."

**Response:** Thank you for your insightful comment. We reviewed Fig. 2 and agree with your opinion that it was closer to a study design than a simulation program setting. Hence, we inserted the figure into the “Design and Setting” section of our manuscript. [Materials and Methods-Design and setting, paragraph 2]

3. Third, along with the Fig. 2 problem, the study design should be more detailed to determine, when the survey was taken, whether there was no difference in simulation experience due to the rotation experience (since for most medical schools in Korea, students’ previous experiences are different due to the rotation assignment). Furthermore, authors should describe the development of simulation programs, whether they adopt previous simulation programs based upon previous studies, etc. It is hard to compare the efficacy of the program if there are any reference points.

**Response:** Thank you for your helpful comment. The students who participated in this study are all studying the same curriculum in one medical school. Therefore, their experience before participating in pediatric clinical practice is also the same. Additionally, since simulation practice is not performed in other clinical departments that rotate at the same time, there is no difference. Furthermore, among various topics related to patient safety, which are important emerging issues, falls were selected as one of the most common accidents in pediatric departments. [Materials and Methods-Simulation program setting, paragraph 1] Although many references were reviewed to set up the simulation program, the program’s contents were developed independently, regardless of these references.

**Reviewer 6**

1. I thought the previous version was acceptable and the current additional revision is acceptable. At some point, the data need to be made available to the readers for scrutiny.

**Response:** Thank you for your affirmative comment. We revised the manuscript based on the comments and suggestions of the reviewers. We hope our manuscript improves further such that it is suitable for publication in your esteemed journal.
